# Metagenomic Sequencing and Quantitative Real-Time PCR for Fecal Pollution Assessment in an Urban Watershed

**DOI:** 10.3389/frwa.2021.626849

**Published:** 2021-02-15

**Authors:** Kyle D. Brumfield, Joseph A. Cotruvo, Orin C. Shanks, Mano Sivaganesan, Jessica Hey, Nur A. Hasan, Anwar Huq, Rita R. Colwell, Menu B. Leddy

**Affiliations:** 1 Maryland Pathogen Research Institute, University of Maryland, College Park, MD, United States; 2 University of Maryland Institute for Advanced Computer Studies, University of Maryland, College Park, MD, United States; 3 Joseph Cotruvo and Associates LLC, Washington, DC, United States; 4 U.S. Environmental Protection Agency, Office of Research and Development, Cincin nati, OH, United States; 5 CosmosID Inc., Rockville, MD, United States; 6 Essential Environmental and Engineering Systems, Huntington Beach, CA, United States

**Keywords:** whole metagenome sequence, fecal indicator bacteria, microbial source tracking, quantitative real-time PCR, metagenomic analysis, culture, rainfall–runoff

## Abstract

Microbial contamination of recreation waters is a major concern globally, with pollutants originating from many sources, including human and other animal wastes often introduced during storm events. Fecal contamination is traditionally monitored by employing culture methods targeting fecal indicator bacteria (FIB), namely *E*. *coli* and enterococci, which provides only limited information of a few microbial taxa and no information on their sources. Host-associated qPCR and metagenomic DNA sequencing are complementary methods for FIB monitoring that can provide enhanced understanding of microbial communities and sources of fecal pollution. Whole metagenome sequencing (WMS), quantitative real-time PCR (qPCR), and culture-based FIB tests were performed in an urban watershed before and after a rainfall event to determine the feasibility and application of employing a multi-assay approach for examining microbial content of ambient source waters. Cultivated *E*. *coli* and enterococci enumeration confirmed presence of fecal contamination in all samples exceeding local single sample recreational water quality thresholds (*E*. *coli*, 410 MPN/100 mL; enterococci, 107 MPN/100 mL) following a rainfall. Test results obtained with qPCR showed concentrations of *E*. *coli*, enterococci, and human-associated genetic markers increased after rainfall by 1.52-, 1.26-, and 1.11-fold log_10_ copies per 100 mL, respectively. Taxonomic analysis of the surface water microbiome and detection of antibiotic resistance genes, general FIB, and human-associated microorganisms were also employed. Results showed that fecal contamination from multiple sources (human, avian, dog, and ruminant), as well as FIB, enteric microorganisms, and antibiotic resistance genes increased demonstrably after a storm event. In summary, the addition of qPCR and WMS to traditional surrogate techniques may provide enhanced characterization and improved understanding of microbial pollution sources in ambient waters.

## INTRODUCTION

Microbiological degradation of surface water used for recreation, i.e., rivers, lakes, streams, and beaches, is a major water quality concern globally. Pathogenic microorganisms contaminating surface water can cause a range of food- and water-borne diseases. In the United States, maintenance and safety of water used for recreation are covered in the United States Environmental Protection Agency (USEPA) Clean Water Act of 1972, which regulates discharge of pollutants into waters and sets water quality standards for surface water (EPA, 2002). However, many U.S. waters fail to meet designated microbiological thresholds of water used for drinking and recreation due to high levels of fecal contamination.

Sources of surface water fecal contamination typically include wastewater treatment plants, septic systems, domestic and wild animal feces, and municipal sewer overflows after heavy rainfall events. Stormwater runoff can lead to surface water contamination by accumulation of microbiological and chemical pollutants on land during dry weather periods and subsequent transport into nearby waterways. Notable examples of waterborne outbreaks include *Escherichia coli* O157:H7 and *Campylobacter jejuni* in Walkerton, Ontario (Auld et al., 2004), *Salmonella spp*. in Georgia, USA (Lee D. et al., 2019), and *Cryptosporidium spp*. in Milwaukee, USA (Curriero et al., 2001). Thus, characterizing the collective microbial composition of water and identifying potential contaminating sources are priorities for local management groups.

Fecal microbial contamination levels are routinely determined by culturing indicator microorganisms, namely enterococci and *E. coli*, common in fecal waste of humans and other warm-blooded animals. A correlation has been established between many general fecal indicator bacteria (FIB) and gastrointestinal illnesses (Wade et al., 2010). However, culture-based monitoring has limitations, particularly an inability to detect presence of all potential pathogens in surface waters since presence of some enteric microorganisms is poorly correlated with FIB (Harwood et al., 2005; Pusch et al., 2005). FIB are not only shed by humans and other warm-blooded animals, but also cold-blooded animals such as amphibians (Gibb et al., 2017), making it difficult to identify the responsible animal pollution source(s) for targeted remediation. Furthermore, *E*. *coli* and enterococci of non-fecal origin capable of proliferating in the natural environment are now widely recognized and may confound FIB water quality monitoring in some areas (Byappanahalli et al., 2012). Thus, there is a benefit of combining water quality molecular methods that can discern between contaminating sources and track potentially public health relevant targets with traditional methods for FIB monitoring; for review, see (Meays et al., 2004; Hamilton et al., 2020; Mathai et al., 2020).

Molecular methods that target specific genetic regions of a microbial genome have long been suggested to complement culture methods for monitoring FIB. As a result, molecular tools, namely quantitative real-time PCR (qPCR) and digital droplet PCR, that can measure fecal contamination levels rapidly, identify sources of contamination, and track public health relevant targets, such as antimicrobial resistance (AR) genes and specific enteric microorganisms, have been developed (EPA, 2004; Yang et al., 2017; Staley et al., 2018). These methods are useful for profiling factors contributing to non-point source contamination, e.g., humans (Shanks et al., 2009), cows (Shanks et al., 2008), dogs (Rojas et al., 2017), pigs (Mieszkin et al., 2009), horses (Dick et al., 2005), waterfowl (Ohad et al., 2016), gull (Lee et al., 2013), geese, ducks, and chicken (Green et al., 2012). However, detection and enumeration of one or more of these genetic markers by themselves does not provide a sufficiently comprehensive set of information that is needed to identify the full range of microorganisms and AR genes that may be present in surface waters contaminated with fecal waste.

More recently, metagenomic sequencing, coupled with bioinformatics, has gained attention as an effective water quality assessment tool (Wang et al., 2016; Burcham et al., 2019; Acharya et al., 2020; Brumfield et al., 2020a). However, many waterborne microbial surveys have relied upon targeted sequencing (Uyaguari-Diaz et al., 2016; Su et al., 2017; Jin et al., 2018), which generally employ universal PCR primers to amplify hypervariable regions of the 16S rRNA gene to infer taxonomic identification of bacteria and archaea by mapping sequencing reads to genomic databases. By employing whole metagenome sequencing (WMS), the bacterial, archaeal, viral, fungal, and protozoan microbiome community members can be profiled, and in some instances, identified to sub-species taxonomic level (Brumfield et al., 2020b). Profiling the complete microbiome by culture-independent technologies provides an assessment of overall microbial community diversity, which can be used by engineers for the development and optimization of biological systems pertaining to functional processes and nutrient cycling, such as nitrogen and phosphorus removal bacteria; for review, see (Ferrera and Sánchez, 2016). A few studies have used WMS to analyze surface water quality (Shanks et al., 2013; Brown et al., 2015; Fisher et al., 2015; Wu et al., 2018; Hamner et al., 2019; Lee et al., 2020), and microbial community composition following rainfall events remains understudied.

The objective of this pilot study was to demonstrate the feasibility and application of using WMS, host-associated and FIB qPCR, and FIB culture to characterize fecal pollution trends in ambient waters through analysis of samples collected in an urban watershed before and after a rainfall event. Relative abundance (RA) of bacteria, archaea, fungi, protists, and viruses, and carriage of AR associated genes was determined to assess microbiological pollution and provide a comparison with results of traditional water quality methods (FIB culture and host-associated targets). Results showed that added benefit can be achieved by incorporating WMS as a complement to culture and qPCR for water quality monitoring.

## MATERIALS AND METHODS

### Site Description and Sample Collection

**FIGURE 1 |:**
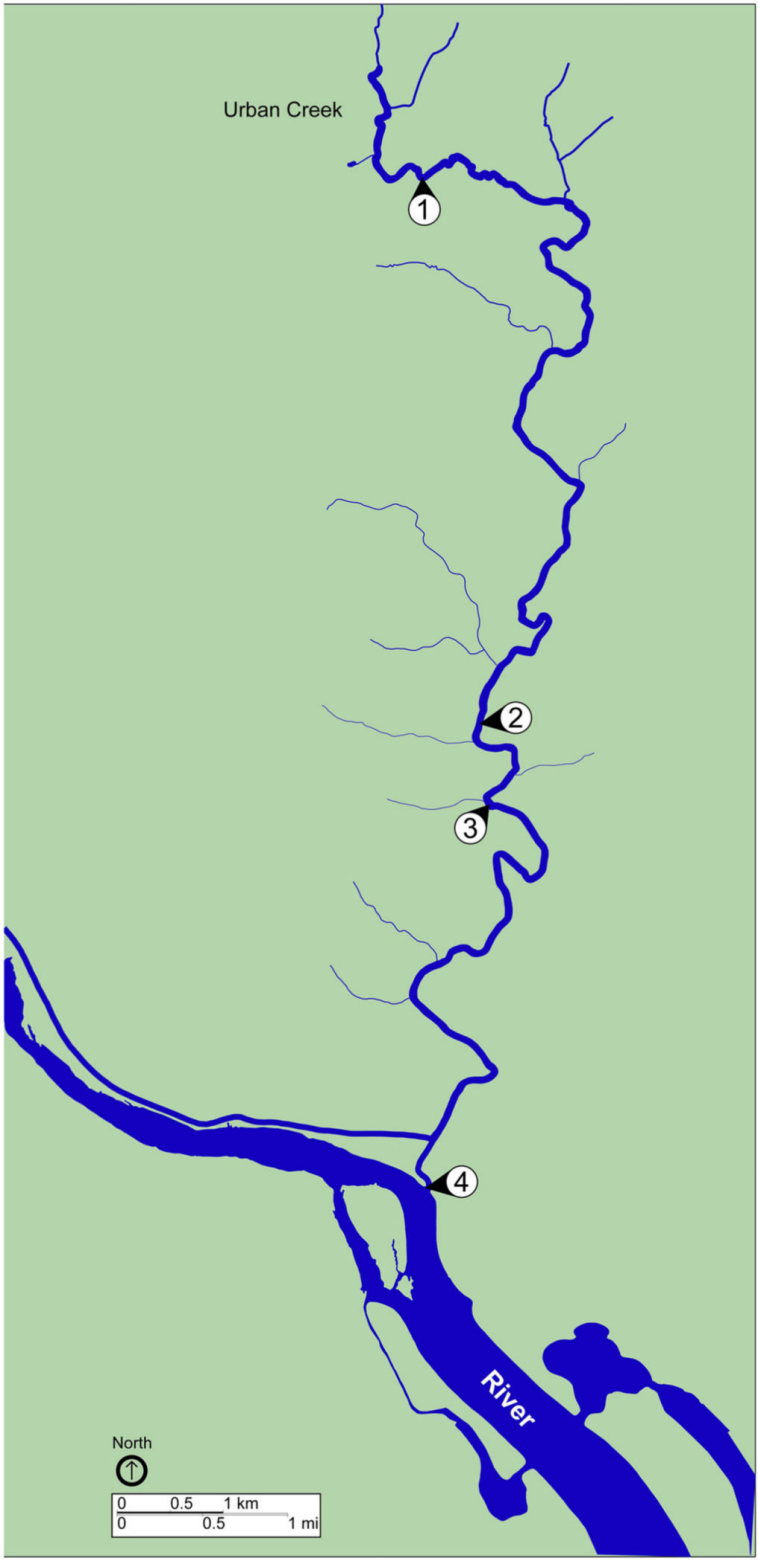
Map of sampling locations. Map shows sites of surface water collection during dry weather, September 23, 2019, and post rainfall, October 24, 2019. Water samples were collected at all four sites during each sampling event. Blue, waterway; green, land. Scale bars are shown to indicate distance.

A total of eight water samples were collected from sites located along a creek in an urban watershed (Figure 1). Sampling sites were consecutively numbered, starting upstream (site 1) and moving downstream (site 4). Grab samples were collected at each site during a dry weather period (no precipitation during the previous week) on September 24, 2019 and ~12 h after the completion of a rain event (42.6 mm total precipitation during the previous 72 h) on October 23, 2019. The sliding 31-day precipitation averages for the study area on September 30 and October 31, 2019 were reported as 0.75 and 4.99 mm, respectively, with the greatest precipitation event (34 mm) occurring on October 16, 2019. Time series of the area-averaged daily precipitation rate for September and October 2019 were reported by Goddard Earth Sciences Data and Information Services Center Interactive Online Visualization and Analysis Infrastructure (Acker and Leptoukh, 2007). Surface water (2 L) was collected at each location using a sterile Nalgene carboy (Thermo Fisher Scientific, Waltham, MA, USA) treated previously with hydrochloric acid (10% v/v), ethanol (95% v/v), and autoclaved. Water samples were transported to the laboratory on ice. Temperature of the water samples was monitored to ensure that it did not reach above 8 °C during transportation by using a LogTag® single trip temperature alert indicator (LogTag Recorders, Auckland, New Zealand). Samples were processed as described below within 2 h of collection and following recommendations of EPA Clean Water Act Analytical Methods (EPA, 2019).

### *Escherichia coli* and Enterococci Surrogate Testing

*Escherichia coli* and enterococci concentrations in the grab samples were enumerated, following manufacturer’s instructions for most probable number (MPN) method per 100 mL of sample water using IDEXX Quanti-Tray System with commercial Colilert and Enterolert media (IDEXX, Westbrook, ME, USA), respectively, providing a culturable bacteria detection range between two and 2,491.6 MPN per 100 mL of water (additional details can be found in the Supplementary Material). During each sampling run, 100 mL of nuclease free water was prepared at site 4 with the Colilert and Enterolert media, respectively, and transported to the lab for processing as mentioned to serve as a trip sterility blank. Cultures of *Enterococcus faecalis* (ATCC® 29212™) and *E. coli* (ATCC® 29212™) obtained from the American Type Culture Collection (ATCC, Manassas, VA, USA) were prepared under standard growth conditions in Luria-Bertani broth at 37 °C overnight (16 h) with aeration, and 100 μl was added to 99.9 mL of nuclease free water containing IDEXX media to serve as positive controls for the Enterolert and Colilert assays, respectively.

### Quantitative Real-Time PCR for Host-Associated and General FIB Genetic Markers

#### Sample Filtration and DNA Purification

At each sampling date and site, 100 mL (dry weather event) and 20 mL (post rainfall event) were filtered in triplicate using 0.45 μm polycarbonate filters (Fisher Scientific, Pittsburg, PA) to capture larger microorganisms. Because dry weather and post rainfall sampling events contained a variable turbidity content, the volume of water filtered was dependent on the filter and when it clogged. Filtrates in sterile 2 mL screw cap tubes containing silica bead mill matrix (GeneRite, North Brunswick, NJ) were shipped on dry ice overnight to the USEPA research laboratory (Cincinnati, OH, USA), and stored at −80°C until DNA purification (< 30 days). DNA purification of filtrates from 24 filters (4 sites × 2 sampling events × 3 replicates/sample) was done using the DNA-EZ RW02 kit (GeneRite LLC, North Brunswick, NJ, USA), as previously described (Li et al., 2019). Three method extraction blanks (MEB) served as controls. DNA extracts were stored in GeneMate Slick low-adhesion microcentrifuge tubes (ISC BioExpress, Kaysville, UT, USA) at 4°C prior to qPCR amplification (< 48 h).

#### Reference DNA Materials

Reference DNA consisted of two plasmid constructs (Integrated DNA Technologies, Coralville, IA, USA) and salmon sperm DNA (Sigma-Aldrich, St. Louis, MO, USA). Plasmid constructs for internal amplification controls (IAC) and calibration standards (all DNA targets in a single construct) were prepared, as previously described (Li et al., 2019). The Reference DNA was stored in GeneMate Slick low-adhesion microcentrifuge tubes (ISC BioExpress, Kaysville, UT, USA) at −20°C.

#### qPCR Amplification

Four host-associated, i.e., human-associated (HF183/BacR287) (USEPA, 2019), ruminant-associated (Rum2Bac) (Mieszkin et al., 2010), canine-associated (DG3) (Green et al., 2014), and avian-associated (GFD) (Green et al., 2012), and two general, i.e., enterococci (Entero1a) (Ludwig and Schleifer, 2000; Siefring et al., 2008) and *E*. *coli* (EC23S857) (Chern et al., 2011), FIB qPCR assays were used in this study along with a sample processing control (SPC) assay (Sketa22) (Shanks et al., 2016), as reported previously. Oligonucleotide sequences used in this study can be found in the supporting information (Supplementary Table 1). All reactions contained either 2 μL of DNA extract or between 10 and 1.0 × 10^5^ target gene copies of reference DNA calibration standards. HF183/BacR287 multiplex reactions also contained 100 copies of IAC template. All reference DNA and water samples were analyzed in triplicate. The log florescence threshold was manually set to either 0.03 (HF183/BacR287, DG3, Rum2Bac, Entero1a, EC23S857, and Sketa22) or 0.08 (GFD). Quantification cycle (Cq) values were exported to Excel (Microsoft, Redmond, WA, USA) for further analysis.

#### Quality Controls

To monitor for potential extraneous DNA contamination during qPCR amplification, six no-template controls (NTC) with purified water substituted for template DNA were performed with each instrument run. SPC protocol was used to identify suitable and consistent DNA recovery from each water sample, as previously described (Shanks et al., 2016). HF183/BacR287 multiplex IAC procedure was used to monitor for amplification inhibition. For each GFD instrument run, a melt curve analysis with a resolution of 0.3°C was used after thermal cycling to identify spurious amplicons that could confound data interpretation (no spurious amplicons detected; data not shown).

#### qPCR Data Analysis

DNA calibration models were generated for each qPCR assay instrument run using the “single” Bayesian Markov Chain Monte Carlo approach (Sivaganesan et al., 2010). Amplification efficiency (*E*) for each reference DNA calibration model was calculated as follows: $E=10^{\left(-\frac{1}{\text{ slope }}\right)}-1$. The lower limit of quantification (LLOQ) was defined as the 95% credible interval upper-bound from repeated measurement (*n* = 3) of 10 copies per reaction reference DNA standard dilution. To investigate the influence of rainfall, water samples were organized into dry weather and post rainfall sample groups (4 sites × 2 sampling events × 3 filters/sampling event × 3 replicates/filter = 36 reactions per sample group). A fecal score ratio can be used to estimate the relative level of host-associated or general fecal contamination present between dry and post-rainfall sampling events based on the weighted average source-specific gene concentration observed in each group. Weighted average fecal score ratios (average log_10_ copies ± 95% Bayesian credible interval) were estimated for each qPCR assay based on rainfall (post rainfall or dry weather) data group definition utilizing all measurements including non-detects (ND), detections below the LLOQ (BD), and measurements within the range of quantification (ROQ), as reported elsewhere (Cao et al., 2018; Shrestha et al., 2020). A sample group was eligible for fecal score ratio determination if each sample group (post rainfall/dry weather) had at least one BD measurement. To account for different sample volumes between post rainfall (20 mL) and dry weather (100 mL) samples, fecal scores were adjusted to indicate a 20 mL test volume (subtraction of log_10_(5) from dry weather fecal scores prior to calculating ratios). All statistical data analyses were conducted using WinBUGS v.1.4.3 (University of Cambridge, 2020), Statistical Analysis Software (SAS Institute, Cary, NC, USA), and Excel (Microsoft, Redmond, WA, USA).

### Whole Metagenome DNA Sequencing

A total of 600 mL of water from each sampling event was concentrated by using a combination of vacuum and syringe filtration. Water samples were first passed through six 0.6 μm pore size 25 mm polycarbonate Whatman Nuclepore Track-Etch Membranes (Millipore Sigma, St. Louis, MO, USA) by vacuum filtration, to remove trace minerals and other particulates and expedite subsequent filtration. Resulting filtrate was collected aseptically and passed consecutively through a single 0.22 μm pore size Sterivex™ Filter Unit (Millipore Sigma, St. Louis, MO, USA) by using syringe filtration and six 0.1 μm pore size 47 mm polycarbonate Whatman Nuclepore Track-Etch Membranes (Millipore Sigma, St. Louis, MO, USA) by vacuum filtration to capture smaller microorganisms, including some viruses and phages. All filter membranes were stored at −80°C until DNA was prepared (< 30 days).

For each sample, total DNA was isolated from the microbial biomass collected on the Sterivex™ Filter Unit using the Qiagen DNeasy Power Water Sterivex Kit (Qiagen, Germantown, MD, USA), following manufacturer’s instructions, to obtain a final elution volume of 60 μl. DNA was prepared from all 12 filter membranes using the Qiagen DNeasy Power Soil Kit (Qiagen, Germantown, MD, USA), with the following modifications for DNA extraction from the filter membranes. The 12 filter membranes for each sample were cut into ribbons ~2 mm by 10 mm and distributed evenly amongst six Power Bead Tubes included in the Qiagen Power Soil Kit (Qiagen, Germantown, MD, USA), and final elution volume for each of the six membrane preparations was 20 μl. Eluted DNA was pooled for each Sterivex™ Filter Unit and the six membrane preparations to achieve 180 μl. DNA was purified using DNA Clean and Concentrator™-25 Kit (Zymo Research, Irvine, CA, USA), following manufacturer’s instructions, providing a final elution volume of 50 μL.

Concentrations of genomic dsDNA were measured using Qubit® dsDNA High Sensitivity Assay Kit (Thermo Fisher Scientific, Waltham, MA, USA) on an Invitrogen Qubit® 4.0 Fluorometer (Thermo Fisher Scientific, Waltham, MA, USA). DNA libraries were prepared using Nextera XT DNA Library Prep Kit (Illumina Inc., San Diego, CA, USA). All DNA libraries were quantified, as previously mentioned, and sequenced using an Illumina HiSeq4000 Instrument (Illumina Inc., San Diego, CA, USA) with a 2 × 150 bp run. A negative sequencing control, consisting of nuclease-free water, and a sequencing standard, i.e., ZymoBIOMICS™ Microbial Community Standard (Zymo Research, Irvine, CA, USA), were included for quality assurance of high-throughput sequencing. Metagenomic samples were sequenced with an average of 50.9 (min = 44.2; max = 56.8) million sequence read depth across samples.

#### Whole Metagenome Sequence Analysis

General sequencing statistics for all samples and mean sequence quality distribution, measured by FastQC (v.0.11.6) (Andrews, 2019), are detailed in Supplementary Table 2. Base-calling error probabilities (P) were evaluated using Phred Quality Score (Q), defined by: *Q* =−10 log_10_(*P*). Using a previously defined read quality threshold (Roy et al., 2018; Brumfield et al., 2020a), read libraries were above a Phred Quality Score of 17 for at least 80% of the read lengths, i.e., probability of correct base call was at least 98%, so the reads were not subjected to quality trimming. The average Illumina sequencing read lengths across all libraries was 151 bp.

Unassembled metagenomic sequencing reads were analyzed, as described previously (Lax et al., 2012; Ponnusamy et al., 2016; Roy et al., 2018; Connelly et al., 2019; Brumfield et al., 2020a), using CosmosID Metagenomics Cloud Application v.1.0 (Cosmos ID, 2019) to achieve multi-kingdom microbiome analysis and profiling of AR associated genes and quantification of the organism RA, defined as the proportion of unique organism-specific k-mers annotated by each database relative to the total number of unique sequencing reads generated for that sample. Additional information on the bioinformatics pipeline employed for taxonomic classification of sequencing reads can be found in the supporting information.

Principal coordinate analysis (PCoA) employing Bray-Curtis distance measure and alpha diversity via CHOA1 index were performed based on RA of bacterial taxa in each sample. Analysis of community resistome was achieved by identifying AR associated genes based on percent coverage as a function of gene-specific k-mer frequency in each sample. Sunburst visualizations of taxonomic composition for each sampling event, were generated using Krona (Ondov et al., 2011).

#### Data Availability

Illumina paired metagenomic sequencing data generated for all samples in this study are deposited in the NCBI Sequence Read Archive database (https://www.ncbi.nlm.nih.gov/sra) under BioProject PRJNA655751. Accession numbers for individual sample sequencing read libraries are provided in the supplementary information.

## RESULTS

### Enumeration of *Escherichia coli* and Enterococci

**FIGURE 2 |:**
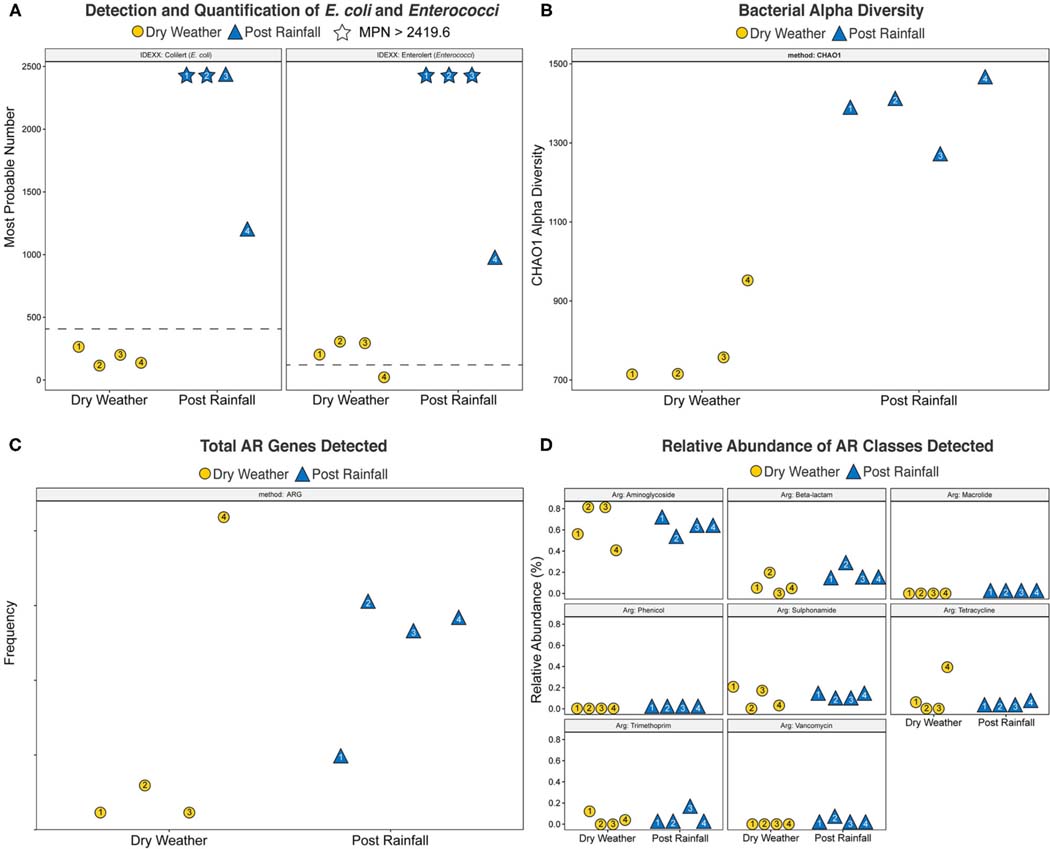
Scatter plots of **(A)** Most Probable Number (MPN) of E. coli and Enterococci generated using the IDEXX Quanti-Tray system with Colilert and Enterolert media, respectively, **(B)** bacterial alpha diversity calculated by CHAO1 index, **(C)** total Antibiotic Resistance (AR) genes detected, and **(D)** relative abundance of AR classes detected. Yellow circles denote a dry weather sampling event; blue triangles indicate a post rainfall sampling event. Number inside each shape corresponds to site location. Plots were generated using the R software package ggplot2 (Wickham, 2016). For **(A)**, dotted line represents USEPA recommended water quality standards for recreational freshwater watersheds in the study area (E. coli, 410 MPN/100mL; enterococci, 107 MPN/100mL); stars indicate that the respective single sampling event yielded > 2,419.6 MPN/100mL.

Concentrations of cultural FIB were determined using the IDEXX/Quanti-Tray FIB assay, and results for each sample are shown in Figure 2A. Within dry weather sampling events, the concentrations of *E*. *coli* ranged between 117.8 and 248.1 MPN/100 mL, and enterococci ranged from 36.8 to 313 MPN/100 mL. During the post rainfall sampling event, variation in the concentration of *E*. *coli* and enterococci could only be assessed qualitatively because dilutions tested yielded results exceeding the upper limit of quantification; that is, concentrations of *E*. *coli* (sites 1 and 2) and enterococci (sites 1–3) in these samples were >2,491.6 MPN/100 mL. Thus, post rainfall, all samples exceeded the local single sample recreational water quality thresholds of 410 MPN/100 mL and 107 MPN/100 mL for *E. coli* and enterococci, respectively.

### Host-Associated and General FIB qPCR

#### qPCR Quality Controls

Calibration model performance parameters (slope and y-intercept parameters, LLOQ, linearity (*R*^2^), and *E*) are provided in Supplementary Table 3. Calibration model *R*^2^-values were ≥ 0.997 and *E*-values ranged from 0.93 (Rum2Bac) to 1.01 (DG3 and GFD). Extraneous DNA control reactions indicated 94.4% were DNA-free. False positives Cq values (*n* = 10) were higher than respective LLOQ in all cases (lowest false positive = 38.3 Cq). Amplification inhibition was absent in all samples based on multiplex IAC HF183/BacR287 testing. IAC acceptance thresholds and competition thresholds ranged from 37.2 to 37.8 Cq and 27.8 to 31.2 Cq, respectively. All 24 water filter DNA extracts passed SPC testing, exhibiting negligible matrix interference. The SPC acceptance threshold was 33.0 Cq. IAC proficiency testing (Run 1 = 0.62 Cq and Run 2 = 0.45 Cq) and SPC proficiency screening (Batch = 0.57 Cq) indicated acceptable consistent implementation of these control experiments.

#### Host-Associated and General FIB qPCR Results

Water quality genetic markers for host-associated and general FIB were measured from 24 filters representing temporal sampling before and after a rain event. Table 1 provides the number of qPCR measurements for each assay organized into ND, BD, and ROQ categories. It is important to note the difference in water sample volumes between dry weather (100 mL) and post rainfall (20 mL) samples. Of the total 432 measurements, 49.8% were ND, 24.8% BD, and 25.5% ROQ. General FIB (Entero1a and EC23S857) accounted for 91.8% of all ROQ (*n* = 101) followed by HF183/BacR287 (*n* = 9). The frequency of ROQ was higher post rainfall (29.2%; *n* = 63) compared to dry weather (21.8%; *n* = 47), despite the fivefold difference in sample volume. Enterococci and *E*. *coli* as well as avian and human host-associated genetic markers were detected in post rainfall and dry weather samples. Ruminant-associated genetic markers were detected in dry weather samples, while dog fecal waste was detected in post rainfall samples. DG3 (dog) was not detected in dry weather large volume samples (100 mL) but were detected in 41.7% of samples post rainfall (20 mL). In addition, Rum2Bac (ruminant) was not detected in dry weather samples; however, Rum2Bac was detected in post rainfall samples.

#### Fecal Score Ratio

**FIGURE 3 |:**
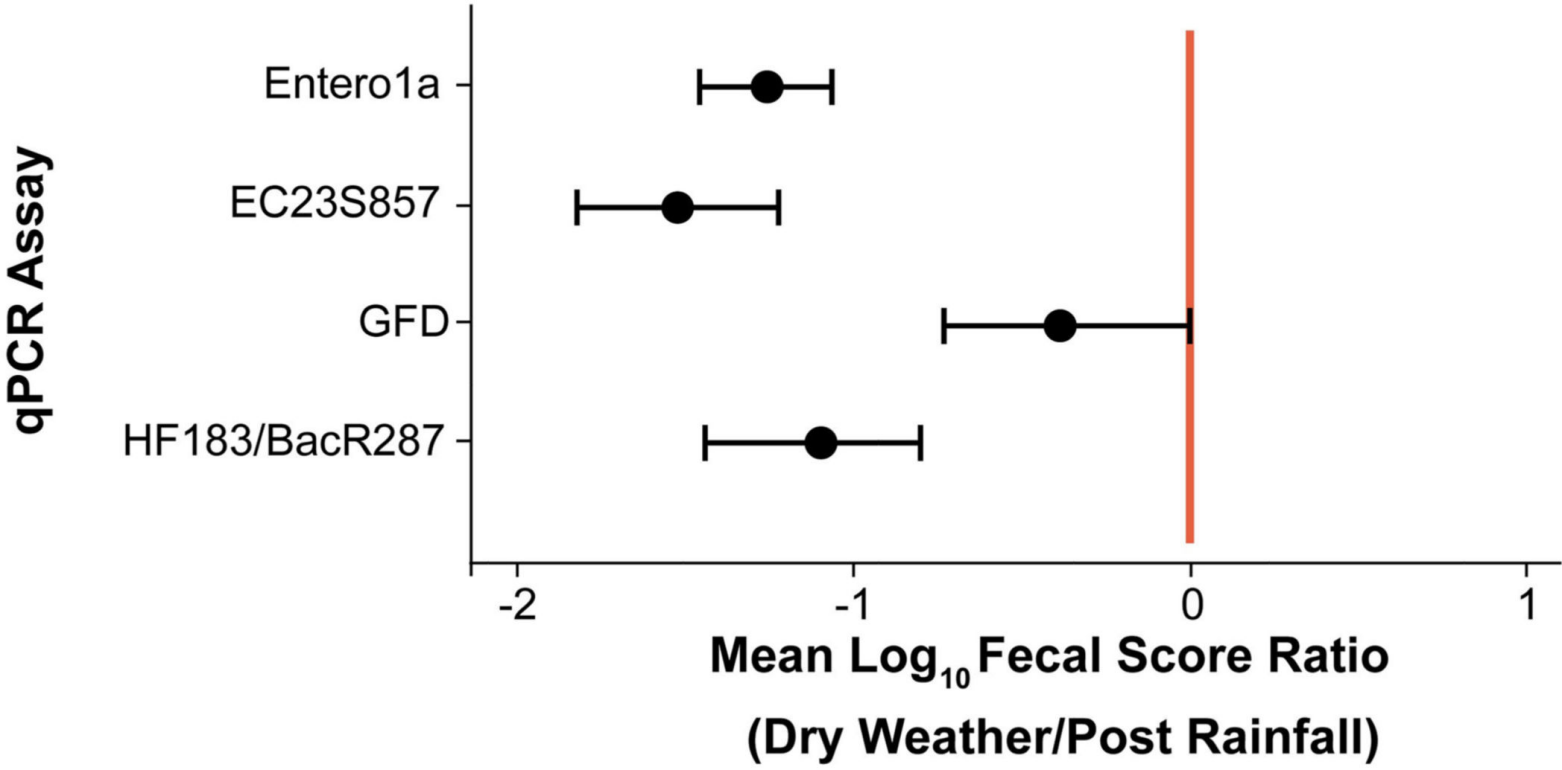
Scatter plot of mean log10 fecal score ratios (Dry Weather/Post Rainfall) and 95% Bayesian Credible Intervals (BCI) for each qPCR assay. Shaded circles represent mean log10 fecal score ratios and error bars depict respective 95% BCI. Vertical red line denotes a log10 fecal score ratio of zero. Mean log10 fecal score ratio values to the left of red line with no interval overlap indicate scenarios of given qPCR assay genetic marker average log10 concentration significantly higher after rainfall (post rainfall).

Due to the large number of ND and BD results (74.5% of total), a censored data fecal score ratio (Cao et al., 2018; Shrestha et al., 2020) was used to calculate weighted average log_10_ copies per reaction 95% Bayesian Credible Interval (BCI) ratio (post rainfall/dry weather) for each eligible qPCR assay data set, which included Entero1a, EC23S857, HF183/BacR287, and GFD qPCR assays (Table 1). Log_10_ fecal score ratios for Entero1a [−1.26 (−1.07 to −1.46 95% BCI)], EC23S857 [−1.52 (−1.22 to −1.82 95% BCI)], and HF183/BacR287 [−1.11 (−0.81 to −1.45 95% BCI)] indicate significantly higher concentrations of fecal sources for post rainfall when compared to dry weather sample groups (Figure 3). However, the avian marker, GFD, exhibited a different trend, with significant difference between post rainfall and dry weather sample fecal scores (95% BCI intersects at 0; −0.73 to 0.01).

### Metagenomic Data Analysis

#### Community Microbiome

**FIGURE 4 |:**
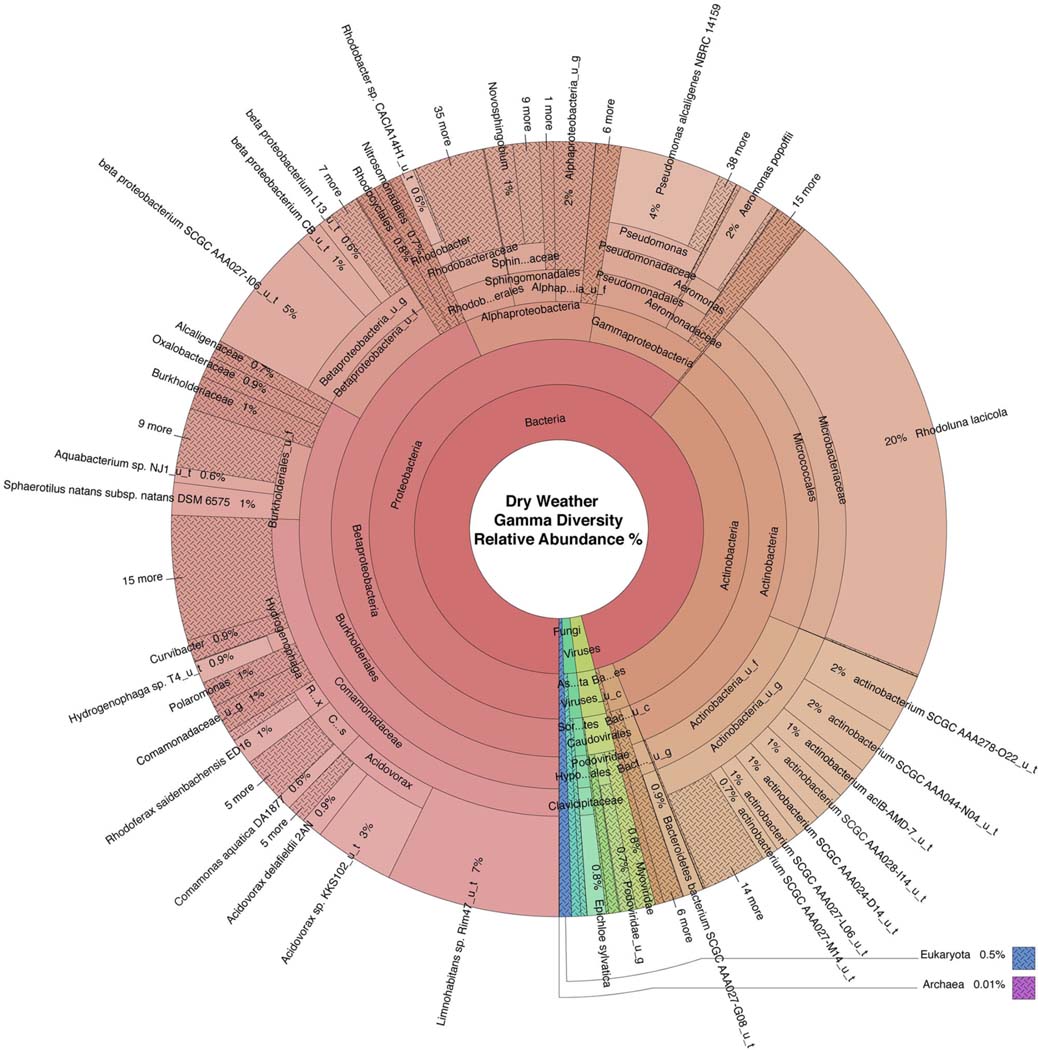
Krona plot of normalized dry weather water microbiome. Species composition percentages are displayed as average number of organism specific k-mers detected, normalized to represent the proportion of organism specific k-mers observed relative to total microbial species diversity detected across samples obtained from all four locations during the dry weather sampling event. Red, bacteria; blue, protozoa; teal, fungi; purple, archaea; green, viruses.

**FIGURE 5 |:**
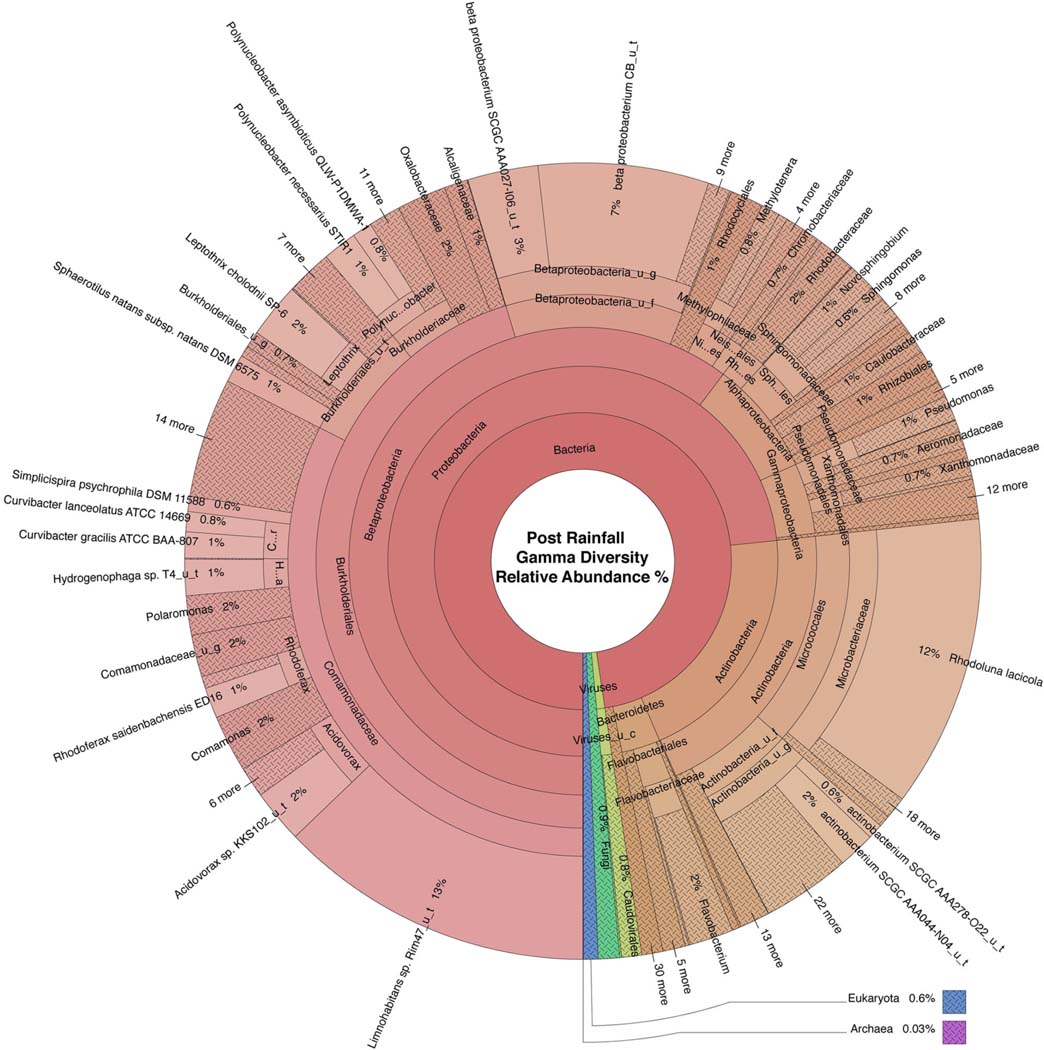
Krona plot of the normalized water microbiome post rainfall. Species composition percentages are displayed as average number of organism specific k-mers detected, normalized to represent the proportion of organism specific k-mers observed relative to total microbial species diversity detected across samples obtained from all four locations during the sampling period following rainfall. Red, bacteria; blue, protozoa; teal, fungi; purple, archaea; green, viruses.

WMS, using DNA prepared from the water samples, generated ~815 million reads across the raw sequence libraries equating to roughly 620 million unique reads (supplementary information). Total bacterial alpha diversity was calculated using CHAO1 index (Figure 2B) and ranged from 712 to 952 in dry weather samples and from 1,273 to 1,468 in samples post rainfall. Bacterial communities in water samples collected during dry weather and post rainfall were analyzed by three-dimensional PCoA using Bray-Curtis dissimilarity index (Supplementary Figure 1), where distance between points indicates degree of difference in bacterial DNA sequence composition. Each sample contained a relatively distinct bacterial composition; however, like samples clustered more closely with like samples, e.g., post rainfall samples clustered more closely compared to those collected during dry weather. Bacteria, archaea, fungi, protozoa, and viruses (including bacteriophages) identified by WMS characterization are shown in Krona plots, representing RA of microbial species gamma-diversity, i.e., total microbial species diversity, detected in dry weather (Figure 4) and post rainfall (Figure 5).

#### Community Resistome

The total number of AR and classes of AR associated genes are shown in Figures 2C,D, respectively. With the exception of site 4, the number of AR genes detected across each of the locations was lower in samples collected during dry weather compared to those collected after rainfall. Furthermore, site 4 was dominated by tetracycline resistance genes that were not detected in the dry weather samples obtained from the other locations but were detected in all samples post rainfall. Overall, the relative abundance of various antibiotic classes detected by WMS did not vary demonstrably across sampling events, and AR genes associated with the aminoglycoside class of antibiotics were dominant in all samples.

#### Wastewater-Associated Enteric Microorganisms

WMS allowed detection of multiple wastewater-associated enteric microorganisms (Table 2). RA of *Aeromonas hydrophilia* was roughly one log greater at all sites following rainfall. *E*. *coli* was detected at all sites at roughly equal RA; however, at site 1 during dry weather sampling, *E. coli* comprised 0.36% RA. *Vibrio cholerae* was detected at all locations, except site 2. *Enterococcus spp*. (*E*. *casseliflavus*, *E*. *faecalis*, and *E*. *faecium*), were detected at sites 1 and 3 during dry weather sampling, while enterococci were detected at all sites except site 3 following rainfall. *Legionella pneumonophilia* was detected at site 4 only following rainfall at extremely low RA. Fast growing *Mycobacteria* (*M*. *chelonae*, *M*. *abscessus*, and *M*. *phlei*) were detected at all locations following rainfall and at site 4 in the dry weather samples. MAC (*M*. *avium* and *M*. *intracellulare*) was detected at 0.03% RA at site 1 and at site 2 (and at lower RA) following rainfall but not detected in any other samples. *M*. *tuberculosis* was detected at 0.02% RA (low abundance) at sites 1 and 3 following rainfall and site 2 before rainfall. Following rainfall, *Yersinia enterocolitica* was detected at low RA at all sites. *Campylobacter coli* was detected only at site 4 in the dry weather samples. *Burkholderia pseudomallei* was detected at all sites following rainfall and detected at sites 3 and 4 in the dry weather samples. Similarly, *Salmonella enterica* was detected at all sites except site 3 following a rainfall and detected at sites 3 and 4 in the dry weather samples. Human *mastadenovirus* C was detected only at site 4 after rainfall. *Cryptosporidium muris* was detected at all post rainfall sites except site 3 and detected at sites 1 and 2 in the dry weather samples. *Acanthamoeba spp*. were detected at all sites and were most abundant at sites 1 and 2 in the dry weather samples.

#### Human-Associated, and Wastewater-Associated FIB

Using a collection of microorganisms identified form the scientific literature, WMS was employed to analyze the microbiomes for general FIB and human- and wastewater-associated microorganisms (Table 3). Generally, FIB were scarce in dry weather samples; however, *Bacteroides spp*. were detected at 0.4% RA at site 4. In contrast, following rainfall, both *Bacteroides spp*. and *Bifidobacterium spp*. were detected at all sites. *Clostridium spp*. were detected at low RA at site 4 after rainfall and at all locations before rainfall. *Citrobacter spp*. were detected at roughly equal RA in all samples except site 3 in dry weather samples. *Escherichia spp*. were most abundant at site 1 in dry weather samples (0.36%) and detected at roughly equal RA at all other locations during dry weather and post rainfall sampling. *Enterobacter spp*. were detected in all samples (except site 2 during dry weather). *Klebsiella spp*. were detected in all samples and were identified at between 0.07 and 0.1% RA post rainfall.

## DISCUSSION

### Fecal Indicator Bacteria

Culturable *E*. *coli* and enterococci were detected in all samples regardless of site (Figure 2A). Furthermore, all samples post rainfall exceeded the USEPA recommended water quality standards for single sample values of recreational freshwater watersheds in the study area (*E*. *coli*, 410 MPN/100 mL; enterococci, 107 MPN/100 mL) (EPA, 2012; DCMR, 2014). A similar trend was observed with FIB identified by qPCR where the indicators were detected in 95.1% of the samples. After rainfall, Enterococci and *E*. *coli* genetic marker weighted average log_10_ concentrations were 1.26- and 1.52-log_10_ copies per 100 mL times higher, respectively. These findings are in agreement with previous studies where precipitation was found to contribute to high concentrations of FIB in surface water (Lee et al., 2020). High FIB concentrations are commonly reported in many urban watersheds throughout the U.S. For example, mean *E*. *coli* concentration of 1,156 MPN/100 mL was reported for a tributary of the Des Moines River in Iowa, USA (Schilling et al., 2009).

Generally, when FIB were detected in post rainfall samples by culture and qPCR, WMS supported the increased RA of FIB in samples collected after rainfall (Table 3). Unlike culture and qPCR methods, taxonomic profiling of metagenomic sequencing reads associated with *E*. *coli* and enterococci indicated comparatively low numbers. The results suggest that WMS may not be suitable for FIB monitoring alone since results can be susceptible to shifts in occurrence associated with changes in RA of other community members. As a result, WMS is best applied as a complement to culture and qPCR by providing useful information toward understanding microbial diversity and occurrence of AR genes, presence of enteric microorganisms, as well as additional information on sources of contamination.

### Host-Associated Fecal Pollution

Identifying sources of fecal pollution in surface waters is a public health challenge, and qPCR is currently employed for surface water quality testing, namely to quantify specific genes in environmental samples (Dick et al., 2005; Shanks et al., 2008, 2009; Mieszkin et al., 2009; Green et al., 2012; Lee et al., 2013; Ohad et al., 2016; Rojas et al., 2017). In the study reported here, the weighted average fecal score ratio (log_10_ copies per 100 mL) of the HF183/BacR287 genetic marker was −1.11 or ~12.8-fold greater after rainfall (Table 1, Figure 3). This indicates sewage and/or other sources of human waste, such as storm drain overflow that can contribute to water quality degradation in the urban watershed. In contrast, the avian-associated GFD genetic marker showed no significant difference between post rainfall and dry weather samples suggesting birds do impact water quality but perhaps independent of rainfall patterns. The ruminant-associated genetic marker (Rum2Bac) was detected in all samples but at very low incidence in this watershed. The canine-associated DG3 genetic marker was detected only in samples of water collected after a rainfall. Most probably surface runoff introduced canine excretory waste into the watershed.

The WMS and bioinformatic annotation strategy employed in this study identified fecal-associated microorganisms with finer taxonomic resolution; this has been observed in an earlier study (Brumfield et al., 2020b). Microbial species reported to be closely associated with the gut microbiota of specific animal groups (Puig et al., 1999; Buchan et al., 2001; Newton et al., 2013; Shanks et al., 2013; Harwood et al., 2017; Jebri et al., 2017) suggest WMS can be used to shed light on potential sources of fecal pollution. Viruses were detected in samples collected after a rainfall, with crAssphage exhibiting a strikingly similar pattern to the HF183/BacR287 qPCR genetic marker, showing a change between dry weather and post rainfall samples. CrAssphage was recently reported as a dsDNA *Bacteroides* bacteriophage tightly associated with human waste (Dutilh et al., 2014) and highly abundant in sewage (Korajkic et al., 2020). Future experiments comparing qPCR (Stachler et al., 2017) and WMS measurements of crAssphage in surface waters are planned to confirm this observation.

### Microbial Diversity of an Urban Watershed

The autochthonous microbiome of surface water is important in sustainability of natural ecosystems, and potential microbial shifts during periods of dry weather and post rainfall can provide useful information to microbial ecology and sources impacting their occurrence. Previous investigation has associated high taxonomic diversity of lotic ecosystems in urban areas with low fecal contamination in those waterways (Paruch et al., 2019). In the current study, increased fecal pollution appeared to be paralleled with higher alpha diversity following rainfall (Figure 2B), and work is underway to confirm the impact of stormwater runoff on fecal pollution and microbial diversity.

Dominant bacterial phyla detected in the urban watershed (Figures 4, 5) were similar to those detected in surface water of other freshwater aquatic systems (Newton et al., 2011; Staley et al., 2013, 2014; Brown et al., 2015; Hamner et al., 2019), i.e., *Proteobacteria* comprised more than 60% RA in both dry weather and post rainfall samples. *Actinobacteria*, a common soil microbe often present in pristine waterbodies (Jenkins et al., 2009; Ghai et al., 2011), was also prominent in the microbiome of all samples examined in this study. The majority of viruses detected in the urban watershed were bacteriophages belonging to three major bacteriophage families, i.e., *Myoviridae*, *Siphoviridae*, and *Podoviridae* (Figures 4, 5), a finding in agreement with other reported freshwater viromes (Mohiuddin and Schellhorn, 2015). *Thaumarchaeota spp*., a group of ammonia-oxidizing archaea detected in urban areas demonstrating high rates of nitrification (Reisinger et al., 2016; Epp Schmidt et al., 2019), were present in most samples in this study. *Nitrospirales* were detected predominantly in samples collected following rainfall. These archaea are considered to be important players in recovery of microbial species composing soil microbiomes following disruptive flooding events (Wang et al., 2019). *Clavaria fumosa*, a fungus, and the protozoan *Pseudoperonospora cubensis*, reported by Lee and colleagues (Lee et al., 2020) who used WMS to define the microbial species composition of residential urban stormwater runoff, were detected in this study.

Most watersheds research currently employs FIB identified by culture methods, and a few investigations have adopted qPCR, primarily for enterococci (USEPA, 2012), to link traditional water quality criteria standard definitions to host-associated qPCR. Rapid qPCR for enterococci and *E*. *coli* offers a shorter sample processing time (< 3 h), compared to traditional FIB culturing (> 18 h). Host-associated genetic markers reliably track common fecal pollution sources, e.g., human, avian, dog, and ruminant, as well as provide quantitative information useful for water quality management. However, these methods were all designed and optimized to detect and quantify a known genetic marker, usually requiring an individual assay to detect each specific gene, without being able to identify uncharacterized microorganisms, i.e., any potential pathogens not yet recognized. Here, use of metagenomic analysis includes thousands of targets including those not yet identified by culture or qPCR expanding the breadth of information regarding the water quality and our knowledge as it relates to the changes in the microbial population.

### Community Resistome and Wastewater-Associated Enteric Microorganisms

WMS has been used to explore AR trends in anthropogenically impacted environments (Karkman et al., 2019) and to detect wastewater-associated enteric pathogens (Stamps et al., 2018), including viruses (Lee S. et al., 2019). Stormwater is considered to promote transmission of AR among bacteria (Di Cesare et al., 2017), as well as introduce enteric pathogens (Ahmed et al., 2018), suggesting occurrence of AR and enteric microorganisms can vary between dry weather and after rainfall in our watershed of interest. In this study, we observed an increase in AR genes detected after a rainfall (Figure 2C). At site 4, which is a location near where the creek debouches into a larger river, there was an abundance of tetracycline resistance genes that were not detected in the dry weather samples. It is possible that the additional AR genes detected at this location were impacted by microorganisms introduced to the creek from the larger river during periods of dry weather. However, the relative abundance of various AR classes detected at each site varied only slightly between sampling events (Figure 2D). These observations are the result of a single grab sample at each location, and additional observations are needed to establish a resistome baseline prior to determining the overall impact that rainfall may have on AR gene composition.

In addition to AR, WMS makes possible detection of multiple wastewater-borne enteric microorganisms without *a priori* knowledge. For example, *Cryptosporidium spp*., detected at three of the four sites after rainfall (Table 2), currently is the leading cause of reported cases of diarrhea linked to human parasites in contaminated water in the U.S. (Gharpure et al., 2019) and are also known to infect animals. Similarly, *Aeromonas hydrophilia*, an opportunistic pathogen causing gastroenteritis and blood infections was detected in all samples and at slightly increased in RA after rainfall (Table 2). These findings may add support to other reports concluding that *Aeromonas hydrophilia* is omnipresent at low abundance in surface water (Poffé and de Beeck, 1991), but at high concentrations in raw wastewater (Shannon et al., 2007).

The source of microbial contamination of urban watersheds is often varied and can originate from farms, animal feed lots, septic tanks, combined sewer overflow, among others, and modes of delivery can introduce multiple fecal sources to the waterway which can make mitigation difficult. A microbial signature approach, to identify fecal pollution in waters off an urban coast of Lake Michigan, USA, used 16S rRNA sequencing to classify sequences to three wastewater-associated bacterial genera (*Acinetobacter*, *Arcobacter*, and *Trichococcus*) and five fecal-associated bacterial families (*Bacteroidaceae, Porphyromonadaceae, Clostridiaceae, Lachnospiraceae*, and *Ruminococcaceae*) (Newton et al., 2013). Newton and colleagues determined the RA of sewer and fecal signatures increased to >2% of the measured surface water bacterial communities following sewer overflow. Similarly, during a metagenomic survey of wastewater in the United Kingdom, the genera *Arcobacter* and *Aeromonas* were identified as predominant fecal pollution indicators (Acharya et al., 2020). In this study, with the exception of *Trichococcus*, these sewer and fecal signatures were detected at varying RA between dry and post-rainfall sampling events (Table 3, Figures 4, 5).

Metagenomic sequencing can be used to detect and identify a wide range of microorganisms, including bacteria, viruses, fungi, and protists, in some cases to sub-species level. However, viability or infectious potential of detected microorganisms requires additional analyses of metabolic activity. Metagenomic analysis via short read lengths limits the information available within a single read, and characterization of sophisticated genomic structures requires assembled genomes for analysis (Ayling et al., 2020). However, the coverage of microorganisms present at lower RA is often not sufficient to obtain *de novo* metagenomic assembled genomes. Until novel metagenomic assembly tools are developed, the taxonomic annotation of unassembled sequencing reads remains a viable option for metagenomic data analysis to conserve as much of the less abundant species sequence as possible (Lax et al., 2012; Ponnusamy et al., 2016; Roy et al., 2018; Connelly et al., 2019; Brumfield et al., 2020a). Direct testing of sediments and potential fecal pollution sources harboring pathogens near the study area could also be helpful to index and further describe the microbial communities associated with stormwater runoff and to differentiate the transient stormwater population from the indigenous microbial population.

## Conclusions

This pilot study demonstrates the feasibility and application of combining traditional and non-conventional techniques to characterize microbial communities in ambient waters and identify potential sources of pollution. FIB by culture, qPCR amplification of FIB and host-associated genetic markers, and WMS to detect, identify, and enumerate bacteria, archaea, fungi, protists, and viruses were employed. This comprehensive strategy provided useful insights of the microbial constituents present during dry weather and post rainfall in the surface water of an urban watershed and could potentially improve water quality management in the U.S. and globally (United Nations, 2018). Trends were characterized with a limited number of grab samples and are illustrative of a single event representing temporal shifts in microbial communities before and after rainfall. Further assessments are warranted to identify the naturally occurring community ecology and establish a comparative baseline that can be used to verify microbial shifts between dry and rain event conditions, explore the influence of antecedent dry period and stream flow, as well as characterize potential public health significance of metagenomic DNA sequence findings. Future stormwater investigations could also shed light on potential shifts in WMS and qPCR results in response to storm size and duration, soil and particulate content, season, groundwater height, elevation, and land use. It could also be useful to compare sample processing, DNA sequencing, and bioinformatic approaches used here with other strategies. It is worth noting that WMS data reporting is typically limited to shifts in the relative abundance of sequence reads. While this practice can provide a wealth of new information, it does not conclude absolute quantification of specific genomic targets of interest, viability, or infectious potential of the detected microorganisms. Furthermore, the use of WMS for monitoring microbial communities can be more expensive and time consuming than traditional techniques and require advanced bioinformatics software for analyses. Future research directions could also include the exploration of inter-method correlation between different microbial water quality indicators (Acharya et al., 2019) and compare different WMS strategies for routine integration of these three methods for water quality monitoring. Until the above future research directions are fully characterized and considered in the context of water management, WMS is best applied as a complement to established culture and qPCR practices.

## Supplementary Material

Supplement1

Supplement2

## Figures and Tables

**TABLE 1 |:** Enumeration using qPCR.

Assay	Determinant	Weather	ND^a^	BD^b^	ROQ^c^	Fecal score ratio eligible
EC23S857	*E. coli*	Dry weather	6	18	12	Yes
		Post rainfall^d^	0	6	30	
Entero1a	Enterococci	Dry weather	1	9	26	Yes
		Post rainfall^d^	0	3	33	
GFD	Avian	Dry weather	22	14	0	Yes
		Post rainfall^d^	30	6	0	
HF183/BacR287	Human	Dry weather	22	5	9	Yes
		Post rainfall^d^	7	29	0	
Rum2Bac	Ruminant	Dry weather	34	2	0	No
		Post rainfall^d^	36	0	0	
DG3	Dog	Dry weather	36	0	0	No
		Post rainfall^d^	21	15	0	

qPCR counts when no-detection (ND) was obtained, detections below LLOQ (BD), and within the range of quantification (ROQ) organized by sampling event type (post rainfall/dry weather) are given.

a no-detection

b below the respective lower limit of quantification.

c measurement within the respective range of qualification.

d sample volumes of 20mL per filter were used.

**TABLE 2 |:** Relative sequencing read abundance of wastewater enteric microorganisms detected in urban watershed microbiomes.

Genus	Species	Illness	Dry weather				After rainfall			
			Site 1	Site 2	Site 3	Site 4	Site 1	Site 2	Site 3	Site 4
Bacteria										
*Aeromonas*	*A. hydrophilia*	Gastroenteritis; Septicemia	<0.01	0.01	0.01	0.01	0.07	0.10	0.10	0.08
*Burkholderia*	*B. pseudomallei*	Melioidosis	-	-	0.03	0.01	<0.01	<0.01	<0.01	<0.01
*Campylobacter*	*Campylobacter spp.*	Gastroenteritis	0.03	0.02	0.02	<0.01	0.02	-	0.03	<0.01
*Yersinia*	*Y. enterocolitica*	Diarrhea, reactive arthritis	-	-	-	-	<0.01	<0.01	<0.01	0.04
*Enterococcus*	*E. casseliflavus*; *E. faecalis*; *E. faecium*	Gastroenteritis	<0.01	-	<0.01	-	<0.01	<0.01	-	0.01
*Escherichia*	*E. coli*	Gastroenteritis	0.36	0.02	0.04	0.02	0.03	0.04	0.02	0.03
*Legionella*	*L. pneumophilia*	Respiratory illness (pneumonia, Pontiac fever)	-	-	-	-	-	-	-	<0.01
*Mycobacteria*	*M. tuberculosis*	Tuberculosis	<0.01	<0.01	-	-	<0.01	-	<0.01	0.02
	*M. avium*; *M. intracellulare*	MAC	-	-	-	-	0.03	<0.01	-	-
	*M. chelonae; M. abscessus; M. phlei*	Extrapulmonary infection	-	-	-	0.01	<0.01	0.02	0.01	0.02
*Salmonella*	*S. enterica*	Gastroenteritis; reactive arthritis	-	-	<0.01	<0.01	<0.01	<0.01	-	<0.01
*Vibrio*	*V. cholerae* (non-O1/O139)	Cholera	0.03	<0.01	-	<0.01	<0.01	<0.01	<0.01	<0.01
**Protozoa**										
*Cryptosporidium*	*C. muris*	Gastroenteritis	1.35	0.57	-	-	0.10	0.10	-	0.23
*Acanthamoeba*	*Acanthamoeba spp.*	Amoebic meningoencephalitis, keratitis, encephalitis	23.66	38.25	1.26	13.89	8.24	14.42	1.94	6.68
**Viruses**										
*Adenoviridae*	Human mastadenovirus C	Gastroenteritis, respiratory illness, eye infection	-	-	-	-	-	-	-	<0.01

Relative abundance (percentage) of unique sequencing reads for each sample, annotated as respective microbial taxa, is shown. Sample RA values are shown as proportion within each kingdom, i.e., bacteria, protozoa, and viruses. Characterization of wastewater enteric microorganisms is defined elsewhere (Poffé and de Beeck, 1991; Shannon et al., 2007; Ramírez-Castillo et al., 2015; WHO, 2017; Richardson and Rautemaa-Richardson, 2019). “-”, not detected; MAC, Mycobacterium avium complex.

**TABLE 3 |:** Relative sequencing read abundance of select general fecal indicators and human-associated microorganisms detected in urban watershed microbiomes.

Associated biomarker	Taxa/Species	References	Dry weather				After rainfall			
			Site 1	Site 2	Site 3	Site 4	Site 1	Site 2	Site 3	Site 4
**Bacteria**										
General FIB	*Bacteroides spp.*	Harwood et al., 2017	-	-	<0.01	0.40	0.03	0.03	0.02	0.05
General FIB	*Bifidobacterium spp.*	Harwood et al., 2017	-	-	-	<0.01	0.01	<0.01	<0.01	0.02
General FIB	*Clostridium spp.*	Harwood et al., 2017	<0.01	0.03	<0.01	0.02	-	-	-	0.02
General FIB	*Citrobacter spp.*	Harwood et al., 2017	<0.01	0.01	-	0.01	0.01	0.01	0.01	<0.01
General FIB	*Escherichia spp.*	Harwood et al., 2017	0.36	0.03	0.04	0.03	0.03	0.05	0.02	0.03
General FIB	*Enterobacter spp.*	Harwood et al., 2017	0.01	-	0.03	0.05	0.06	0.05	0.06	0.08
General FIB	*Klebsiella spp.*	Harwood et al., 2017	0.02	0.04	<0.01	0.02	0.09	0.09	0.10	0.07
Human-associated	*Bacteroides dorei*	Harwood et al., 2017	-	-	-	<0.01	-	-	-	-
Human-associated	*Bacteroides thetiotamicron*	Harwood et al., 2017	-	-	-	<0.01	-	-	-	0.01
Human-associated	*Methanobrevibacter smithii*	Harwood et al., 2017	-	-	-	-	-	-	-	<0.01
Human-associated	*Bifidobacterium adolescentis*	Harwood et al., 2017	-	-	-	<0.01	<0.01	<0.01	<0.01	<0.01
Wastewater-associated	*Acinetobacter spp.*	Newton et al., 2013	0.21	0.09	0.09	0.56	0.60	0.50	0.71	0.57
Wastewater-associated	*Arcobacter spp.*	Newton et al., 2013	0.10	0.03	0.10	0.06	0.05	0.27	0.02	0.18
**Phages**										
General FIB	Somatic Coliphage-*Inoviridae*	Jebri et al., 2017	-	-	-	-	0.10	0.17	-	0.06
General FIB	Somatic Coliphage-*Myoviridae*	Jebri et al., 2017	21.10	10.24	12.46	17.88	12.78	15.30	12.06	10.04
General FIB	Somatic Coliphage-*Podoviridae*	Jebri et al., 2017	13.70	13.17	5.77	45.95	15.01	15.89	11.16	15.96
General FIB	Somatic Coliphage-*Siphoviridae*	Jebri et al., 2017	64.54	71.87	78.37	35.14	70.92	67.39	74.29	72.80
Human-associated	crAssphage	Kongprajug et al., 2019	-	-	-	0.05	0.43	0.16	0.41	0.11
Human-associated	*Bacteroides* *ϕ*B124-14	Ogilvie et al., 2012	-	-	-	-	-	0.21	-	-

Relative abundance (percentage) of unique sequencing reads for each sample, annotated as respective microbial taxa. Sample RA values represent the proportion within each kingdom, i.e., bacteria and bacteriophages. “-”, not detected.
