# Supplementary material for: Metagenomic Sequencing and Quantitative Real-Time PCR for Fecal Pollution Assessment in an Urban Watershed
Source: Front Water. Author manuscript; Available in PMC 2022 Feb 1. (PMC8274573; doi:10.3389/frwa.2021.626849)
Supplement: Supplement2 [file NIHMS1677047-supplement-Supplement2.docx]

Supplementary Material

# 1 IDEXX Colilert and Enterolert Assays

# 1.1 Colilert

IDEXX Colilert assay is a commercial version of the 9223 Enzyme Substrate Coliform Test (Palmer et al., 2018) that utilizes hydrolysable substrates (chromogenic and fluorogenic) for the detection of *Escherichia coli* enzymes. Under this assay, *E. coli* are defined as bacteria that possess β-D-galactosidase, which cleaves the chromogenic substrate to release chromogen, and β-glucuronidase, which cleaves a fluorogenic substrate to release fluorogen. The fluorescent product can be viewed under long-wavelength (365-nm) ultraviolet light and indicates a positive test for *E. coli*. It is important to note that some strains of *Shigella* and *Salmonella* spp., which are considered to be overt human pathogens, may also produce a positive fluorescence response under this assay. Therefore, additional testing approaches should be included to identify contaminating sources.

**1.2 Enterolert**

IDEXX Enterolert assay is a commercial version of the 9230 C Flurogenic Substrate Enterococcus Test (Noble et al., 2018) that uses a hydrolysable substrate to detect enterococci. The substrate media allows enterococci to express β-D-galactosidase while preventing non-enterococci from producing detectable fluorescence.

# 2 Taxonomic Classification of Whole Metagenome Sequencing Reads

Unassembled metagenomic sequencing reads, were analyzed as described previously (Lax et al., 2012; Ponnusamy et al., 2016; Roy et al., 2018; Connelly et al., 2019; Brumfield et al., 2020a), using CosmosID Metagenomics Cloud Application v.1.0 (CosmosID, 2019) to achieve multi-kingdom microbiome analysis and profiling of AR associated genes and quantification of the organism RA, defined as the proportion of unique organism-specific k-mers annotated by each database relative to the total number of unique sequencing reads generated for that sample. Briefly, the application utilizes GenBook^®^, a series of curated reference databases, composed of over 150,000 microbial genomes and gene sequences representing over 15,000 bacterial, 5,000 viral, 250 protozoan, and 1,500 fungal species, as well as over 5,500 AR and virulence-associated genes. The pipeline comprises separate pre-computation and per-sample, computational comparator phases. The pre-computation phase requires a reference microbial database, i.e., GenBook^®^, as input, and output is a whole genome phylogenetic tree, together with sets of variable-length k-mer fingerprints (biomarkers) that are uniquely identified with distinct branches, nodes, and leaves of the tree. The per-sample, computational phase employs edit distance-scoring techniques, similar in function to BLAST, to compare sequencing reads against the fingerprint sets to provide composition and relative abundance estimates at all branches, nodes, and leaves of the tree. Aggregation statistics are used to maintain overall classification precision. The first comparator phase identifies reads for which there is an exact match with a k-mer uniquely identified in one or a set of reference strains/genes; the second comparator then statistically scores the entire read against the reference to verify that the read is indeed uniquely identified with that set. For each sample, the reads are assigned to the strain/gene with the highest aggregation statistics.

# 3 Supplementary Tables and Figures

**3.1 Tables**

**Table S1. qPCR Oligonucleotide Sequences Used in the Study.** F, forward primer; R, reverse primer.

| **Assay** | **Primers (5’ 🡪 3’)** | **Probe (5’ 🡪 3’)** | **Reference** |
| --- | --- | --- | --- |
| HF183/BacR287 | F: ATCATGAGTTCACATGTCCG  R: CTTCCTCTCAGAACCCCTATCC | [FAM]CTAATGGAACGCATCCC[MGB] | (Green et al., 2014a) |
| Rum2Bac | F: ACAGCCCGCGATTGATACTGGTAA  R: CAATCGGAGTTCTTCGTGAT | [FAM]ATGAGGTGGATGGAATTCGTGGTGT[TAMRA] | (Mieszkin et al., 2010) |
| DG3 | F: TGAGCGGGCATGGTCATATT  R: TTTTCAGCCCCGTTGTTTCG | [FAM]AGTCTACGCGGGCGTACT[MGB] | (Green et al., 2014b) |
| GFD | F: TCGGCTGAGCACTCTAGGG  R: GCGTCTCTTTGTACATCCCA |  | (Green et al., 2012) |
| EC23S857 | F: GGTAGAGCACTGTTTTGGCA  R: TGTCTCCCGTGATAACTTTCTC | [FAM]TCATCCCGACTTACCAACCCG[TAMRA] | (Chern et al., 2011) |
| Entero1a | F: GAGAAATTCCAAACGAACTTG  R: CAGTGCTCTACCTCCATCATT | [FAM]TGGTTCTCTCCGAAATAGCTTTAGGGCTA[TAMRA] | (Siefring et al., 2008) |
| Sketa22 | F: GAGAAATTCCAAACGAACTTG  R: CAGTGCTCTACCTCCATCATT | [FAM]TCATCCCGACTTACCAACCCG[TAMRA] | (Haugland et al., 2010) |

**Table S2.** **Sequencing Accession Numbers and Statistics for Each Sample Included in the Study as Measured by FastQC** (Andrews, 2019)**.** Excel file.

**Table S3. Host-associated and General Fecal Indicator Bacteria qPCR Calibration Model Performance Parameters.**

| **Assay** | **Type** | **Slope** | | **Y-Intercept** | | **LLOQ**^b^ | **R^2^** ^c^ | ***E***^d^ |
| --- | --- | --- | --- | --- | --- | --- | --- | --- |
|  |  | *Estimate* | *StDev*^a^ | *Estimate* | *StDev*^a^ |  |  |  |
| DG3 | Dry Weather | -3.30 | 0.04 | 37.20 | 0.13 | 34.11 | 0.998 | 1.01 |
|  | Post Rainfall | -3.33 | 0.02 | 37.19 | 0.08 | 33.99 | 0.999 | 1.00 |
| EC23S857 | Dry Weather | -3.39 | 0.02 | 38.04 | 0.07 | 34.77 | 1.000 | 0.97 |
|  | Post Rainfall | -3.31 | 0.03 | 37.84 | 0.10 | 34.68 | 0.999 | 1.00 |
| Entero1a | Dry Weather | -3.35 | 0.03 | 37.76 | 0.09 | 34.56 | 0.999 | 0.99 |
|  | Post Rainfall | -3.41 | 0.04 | 37.93 | 0.12 | 34.72 | 0.999 | 0.96 |
| GFD | Dry Weather | -3.37 | 0.02 | 35.16 | 0.08 | 31.92 | 0.999 | 0.98 |
|  | Post Rainfall | -3.31 | 0.03 | 34.94 | 0.10 | 31.79 | 0.999 | 1.01 |
| HF183/BacR287 | Dry Weather | -3.45 | 0.05 | 38.13 | 0.15 | 34.92 | 0.998 | 0.95 |
|  | Post Rainfall | -3.41 | 0.02 | 38.08 | 0.08 | 34.80 | 0.999 | 0.96 |
| Rum2Bac | Dry Weather | -3.38 | 0.03 | 40.91 | 0.09 | 37.67 | 0.999 | 0.98 |
|  | Post Rainfall | -3.50 | 0.05 | 41.15 | 0.18 | 37.93 | 0.997 | 0.93 |

^a^ standard deviation

^b^ lower limit of quantification determined from the 95% credible interval upper-bound at 10 copies per reaction

^c^ the linearity of respective calibration model

^d^ amplification efficiency (E = 10^(-1/slope)^ – 1).

**3.2 Figures**


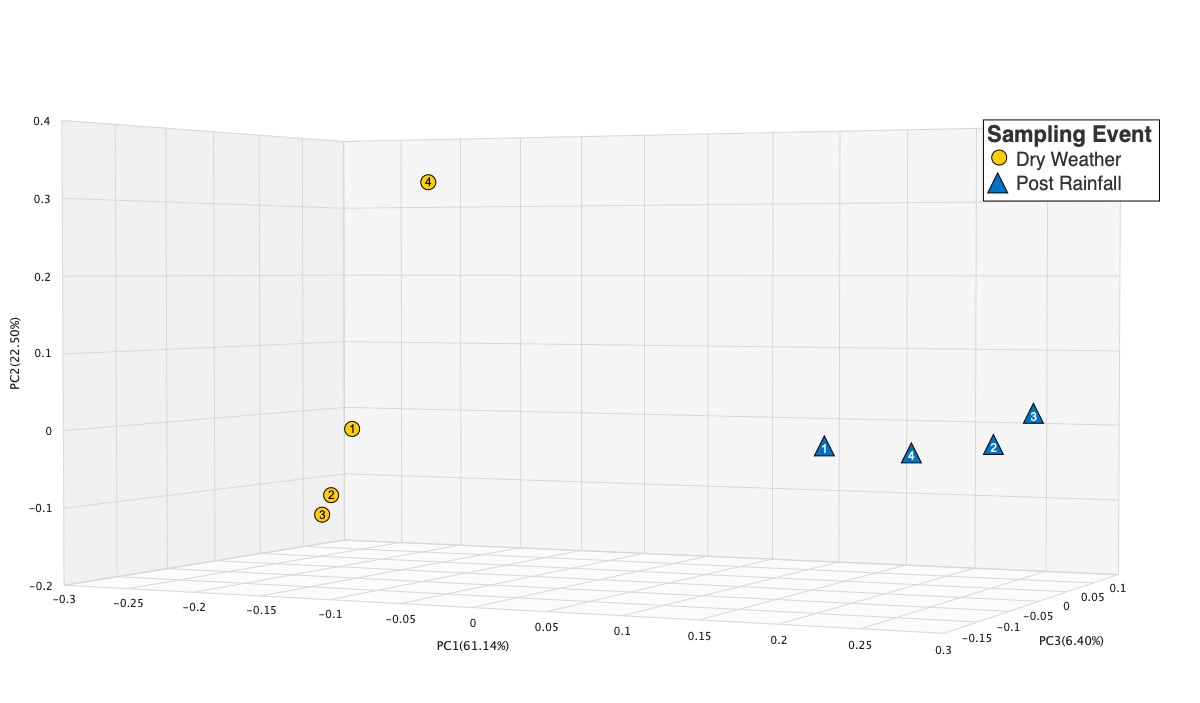


**Figure S1.** **Principal Coordinate Analysis of Bacterial Communities.** Water samples were categorized into clusters by PCoA using the Bray-Curtis distance metric based on relative abundance of bacterial species. Distance between points indicates degree of dissimilarity in bacterial composition, ranging from zero (samples share the same species abundances) to one (samples contain completely different species abundances). The percent variation explained by each axis is indicated. Dry weather and post rainfall samples clustered with like samples. Yellow circle: dry weather sampling event; blue triangle: post rainfall sampling event. Number corresponds to site location.

**4 References**

Andrews, S. C. (2019). FastQC. Available at: https://github.com/s-andrews/FastQC [Accessed October 2, 2019].

Chern, E. C., Siefring, S., Paar, J., Doolittle, M., and Haugland, R. A. (2011). Comparison of quantitative PCR assays for Escherichia coli targeting ribosomal RNA and single copy genes. *Lett. Appl. Microbiol.* 52, 298–306. doi:10.1111/j.1472-765X.2010.03001.x.

Green, H. C., Dick, L. K., Gilpin, B., Samadpour, M., and Field, K. G. (2012). Genetic markers for rapid PCR-based identification of gull, Canada goose, duck, and chicken fecal contamination in water. *Appl. Environ. Microbiol.* 78, 503–510. doi:10.1128/AEM.05734-11.

Green, H. C., Haugland, R. A., Varma, M., Millen, H. T., Borchardt, M. A., Field, K. G., et al. (2014a). Improved HF183 quantitative real-time PCR assay for characterization of human fecal pollution in ambient surface water samples. *Appl. Environ. Microbiol.* 80, 3086–3094. doi:10.1128/AEM.04137-13.

Green, H. C., White, K. M., Kelty, C. A., and Shanks, O. C. (2014b). Development of rapid canine fecal source identification PCR-based assays. *Environ. Sci. Technol.* 48, 11453–11461. doi:10.1021/es502637b.

Haugland, R. A., Varma, M., Sivaganesan, M., Kelty, C., Peed, L., and Shanks, O. C. (2010). Evaluation of genetic markers from the 16S rRNA gene V2 region for use in quantitative detection of selected Bacteroidales species and human fecal waste by qPCR. *Syst. Appl. Microbiol.* 33, 348–357. doi:10.1016/j.syapm.2010.06.001.

Mieszkin, S., Yala, J.-F., Joubrel, R., and Gourmelon, M. (2010). Phylogenetic analysis of Bacteroidales 16S rRNA gene sequences from human and animal effluents and assessment of ruminant faecal pollution by real-time PCR. *J. Appl. Microbiol.* 108, 974–984. doi:10.1111/j.1365-2672.2009.04499.x.

Noble, R., Dichter, G., Genthner, F., McLellan, S., and Solo-Gabriele, H. (2018). “9230 FECAL ENTEROCOCCUS/STREPTOCOCCUS GROUPS (2017),” in *Standard Methods For the Examination of Water and Wastewater* Standard Methods for the Examination of Water and Wastewater. (American Public Health Association). doi:doi:10.2105/SMWW.2882.197.

Palmer, C., Covert, T., Grant, R., Hall, N., Rice, E., Roll, B., et al. (2018). “9223 ENZYME SUBSTRATE COLIFORM TEST (2017),” in *Standard Methods For the Examination of Water and Wastewater* Standard Methods for the Examination of Water and Wastewater. (American Public Health Association). doi:doi:10.2105/SMWW.2882.194.

Siefring, S., Varma, M., Atikovic, E., Wymer, L., and Haugland, R. A. (2008). Improved real-time PCR assays for the detection of fecal indicator bacteria in surface waters with different instrument and reagent systems. *J. Water Health* 6, 225–237. doi:10.2166/wh.2008.022.
